# Concurrent high-intensity interval training and probiotic supplementation improve associative memory via increase in insulin sensitivity in ovariectomized rats

**DOI:** 10.1186/s12906-023-04097-3

**Published:** 2023-07-24

**Authors:** Zeinab Bayat, Arsalan Damirchi, Meysam Hasannejad-Bibalan, Parvin Babaei

**Affiliations:** 1 Department of exercise physiology, Faculty of Physical Education &sport sciences, The University of Guilan, Rasht, Iran; 2 Cellular and Molecular Research Center, School of Medicine, Guilan University of Medical Sciences, Rasht, Iran; 3 Department of Microbiology, School of Medicine, Guilan University of Medical Sciences, Rasht, Iran; 4 Neuroscience Research center, School of Medicine, Guilan University of Medical Sciences, Rasht, Iran; 5 Department of Physiology, School of Medicine, Guilan University of Medical Sciences, Rasht, Iran

**Keywords:** Adiponectin, High-intensity interval training, Learning, Memory, Metabolic syndrome, Probiotic, Ovariectomy

## Abstract

**Objectives:**

Metabolic syndrome (MetS) is a serious concern among postmenopausal women which predisposes them to cardiovascular and cognitive disorders. Healthful diet and exercise training have been essential strategies to prevent the progress of MetS. The aim of this study was to evaluate the effect of supplementation with a native potential probiotic and high-intensity interval training (HIIT) for 8 weeks on retention of associative memory in rats with ovariectomy- induced metabolic syndrome.

**Method:**

Thirty-two female ovariectomized Wistar rats were divided into four groups (n = 8/group): Control (OVX + Veh), exercise (OVX + Exe), probiotic (OVX + Pro), exercise with probiotic (OVX + Exe + Pro). One sham surgery group was included as a control group. Animals received 8 weeks interventions, and then were tested in a step through passive avoidance learning and memory paradigm, to assess long term memory. Then serum levels of adiponectin, insulin and glucose were measured by ELISA and colorimetry respectively. Data were analyzed by Kruskal-Wallis, Mann-Whitney and also One-way analysis of variance (ANOVA).

**Results:**

Eight weeks of HIIT and probiotic supplementation caused an increase in step through latency and shortening of total time spent in the dark compartment in OVX + Exe + Pro group compared with OVX + Veh group. Also significant increase in serum adiponectin levels, in parallel with a reduction in glucose, insulin and HOMA-IR were achieved by the group of OVX + Exe + Pro.

**Conclusion:**

The present study indicates that HIIT combined with probiotics supplementation for 8 weeks effectively improves associative memory in MetS model of rats partly via improving insulin sensitivity and adiponectin level.

## Introduction

As the number of older adults increases, the prevalence of cognitive decline and Alzheimer’s disease(AD) are multiplied, particularly among women over 60 years age [1, 2] due to female sexual hormones senescence [3]. Estrogen as a main female neuroprotective steroid [4, 5] has been reported to facilitate learning and memory [6], while, estrogen withdrawal results in metabolic, cardiovascular [7] and neurodegenerative disorders [8].

Epidemiological studies confirm that menopause is associated with obesity, metabolic syndrome (MetS), hypertension, insulin resistance (IR), and dyslipidemia that all represent risk factors for type 2 diabetes (T2DM) and AD [9, 10]. Similar studies on animal models reported that ovariectomy results in a dramatic reduction in circulating estrogen levels, like naturally occurring menopause and increases visceral fat and insulin resistance in addition to the impairment in both associative [11] and spatial memories [12].

Currently, approved drugs for treatment of AD include memantine and donepezil [13], which only provide a symptomatic relief [14]. It seems that one of the safest strategies to extend women lifespan and slow down the progression of AD is regular physical activity [15]. Exercise has been known to improve brain circulation and plasticity, however response to exercise is not equal in all individuals and seems to be dependent on physiological status of the body such as genetics, hypo-vitaminosis, [16–18], microbiota and other possible unknown factors.

Recently gut microbiota - brain axis describes a bidirectional communication between brain and gut by which gut influences on brain function and behavior, and also brain modulates gastrointestinal tract [19, 20]. It is assumed that normal function of brain depends on natural composition of the gut flora, and dysbiosis might lead to neurodegenerative diseases such as AD [20]. Accordingly, modulating gut microbiota with probiotics has been considered as a possible strategy for AD treatment. Probiotics are microorganisms which interact with gut microbiota and exert beneficial effects on humans and animals [21].

Therefore, in the present study, we hypothesized that consumption of native potential probiotic bacteria together with HIIT might alleviate cognitive deficits. To approach this, we induce menopausal standard model of MetS induced by ovariectomy [22], then animals underwent particular treatments for 8 weeks. Memory deficit, and MetS components were measured by passive avoidance step through paradigm, and biochemical assays respectively. Adiponectin which is a protein secreted by adipose tissue with anti-diabetic, anti-atherogenic, anti-inflammatory [23], and neuroprotective properties [24] was measured by Enzyme-linked immunosorbent assay (Elisa).

## Materials and methods

### Animals

Forty adults female Wistar rats (3 months’ age and 200 g weight) were used in this study. Animals were housed four per cage in a room with temperature of 22° C ± 2 °C, and 12/12-h light/dark cycle (light on 07:00) and fed standard-pellet rat chow and tap water ad libitum. All methods were carried out in accordance with the Sport Sciences Research Institute of Iran Ethics committee; (approved code: IR.SSRI.REC.1400.1087), and protocol adheres to the ARRIVE guideline and National Institutes of Health guide for the care and use of laboratory animals.

### Ovariectomy surgery

**Fig. 1:**
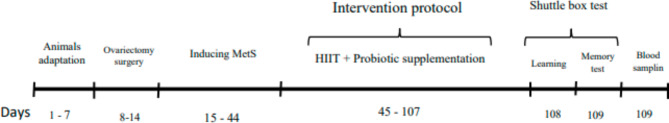
Design of study: adaptation of animals to the new environment for one week in order to acclimate to apparatus and stress high-intensity interval training (HIIT with intensity of 90 to 95% VO_2max_ and zero-degree gradient for three sessions/ week for 8 weeks), and consumption of probiotic (received oral consumption (by gavage syringe) of 1 ml cocktail of Lactobacillus species consisting of 10^9^cells, for three times per week for 8 weeks) of shuttle box Test: Passive avoidance learning Test; Blood sampling: 48 h after the last exercise session

Rats were ovariectomized under general anesthesia with an intraperitoneal (i.p) injection of ketamine (60 mg/Kg) and xylazine (20 mg/Kg) in a ratio of 5:1 [25], and then ovaries bilaterally were removed through single midline incision according to our previous study [26]. The sham group had the similar surgical procedure without removal of ovaries. The design of study was illustrated in Fig. 1.

### Groups and intervention protocol

One month after ovariectomy which estrogen dramatically declines [26], animals were randomly divided into the following groups (n = 8/group): exercise (OVX + Exe), probiotic (OVX + Pro), and combination of exercise with probiotic (OVX + Exe + Pro), Vehicle group (OVX + Veh). Exercised groups ran on a 5 line rodent treadmill (Danesh Salar Iranian Company- DSI-580, Tehran,Iran), with intensity of 90 to 95% VO_2max_ and zero-degree gradient for three sessions/ week. Probiotic groups received oral consumption (by gavage syringe) of 1 ml cocktail of Lactobacillus species consisting of 10^9^cells for three times per week. The control group (OVX + Veh) received the same volume of water by gavage syringe (Fig. 1). Sham control group kept routine life with no intervention.

### Preparation of Lactobacillus strains

According to the previous studies indicating efficacy of Lactobacilli cocktail in comparison with a single strain[27, 28], here we used the native Lactobacilli cocktail isolated from fecal samples in our previous cross-sectional study [29]. Samples were taken from volunteer’s who were referred to rural health centers by convenience sampling method. Inclusion criteria were lack of gastrointestinal diseases and antibiotics consumption during the last six months’ period before sample collection. The probiotic properties of the employed Lactobacillus strains used in this work included bacteriocins production and bactericidal effects, tolerance to acid and bile [29]. In order to perform the test, An MRS agar plate was streaked for isolation from the glycerol bacterial stocks and incubated the plates at 37 ° C for 48 h to ensure purity. A single colony was cultured in MRS broth and after 48 h, the final density of strains at 10^9^ CFU ml − 1 was prepared. Finally, each rat received 1ml of 10^9^ colony forming units/ml (three times per week through the gavage syringe of Lactobacilli cocktail [30].

### High-intensity interval training program

Animals were placed on a rodent treadmill and ran for 10 min at the speed of 10 m/min with a zero-degree gradient for 1 week, in order to acclimate to apparatus and stress. Then they continued running for 8 weeks (3 sessions per week) with intensity of 90 to 95% maximum oxygen consumption (VO_2max_) which followed by one-minute running with intensity of 50% VO_2max_ between 9 intervals. Also, the protocol of warm up (at the beginning of the trainings) and cool down (at the end of the trainings) consisted of 4 min running with an intensity of 55% VO_2max_. To measure the maximum oxygen consumption (VO_2max_), rats ran on treadmill at a speed of 6 m/min with a zero-degree gradient for 5 min (for warm up), and then every 3 min the speed of the treadmill, increased to 3 m/min until the animal reached the level of exhaustion [31].

### Step through passive avoidance learning test

Animals individually were handled in the experiment room for 5 days to acclimate with new arena. Then they were trained in a step through passive avoidance apparatus which consisted of one dark and one light compartment separated by a guillotine door, and stainless-steel shock grid located in the floor of the dark compartment.

At the initial of the experiment, rat was individually transferred to the light compartment and start to explore the new environment for 20 s, then the door between the two compartments was opened, and whenever, the rat entered completely to the dark compartment, an electric foot shock (0.5 mA, 100 Hz/ 2–3 s) was delivered to the floor grids and rat escaped to the light room and immediately returned to its home cage by experimenter. To evaluate long term memory, next day, the rat was placed in the bright room while the door between the two chambers was open, and no shock was delivered. In day 1(learning paradigm), the entrance latency to the dark part of step-through (STL1) was recorded, and in the second day besides STL2, total time spent in the dark section (TSD), and also frequency of entering were recorded for 300 s [11, 32, 33].

### Biochemical measurements

After the end of behavioral test, animals were deeply anesthesized with an intraperitoneal (i.p) injection of ketamine (60 mg/Kg) and xylazine (20 mg/Kg) in a ratio of 5:1 [25], and blood samples were taken, and centrifuged to obtain serum. After blood sampling from vena cava (almost 5ml) animals were sacrificed. Serum adiponectin and insulin were measured by ELISA using Rat-Adiponectin/Acrp30, DY1119, ELISA kit, USA & Canada, and Insulin kit, DRG, USA, Cat. No: DEIA1897. Serum glucose determined by enzymatic colorimetric method (GOD-PAP, Tehran, Iran). Also insulin resistance index (IRI) was assessed by homeostasis model assessment of insulin resistance (HOMA-IR) according to the following formula:

Fasting insulin (µU/mL) × fasting glucose(mmol/L) / 22.5.

### Statistical analysis

Variables from the passive avoidance learning part were evaluated by Kruskal-Wallis test and non-parametric Mann-Whitney test in SPSS ver 22 (USA). Also one-way analysis of variance (ANOVA) and post hoc Tukey tests were used for group comparisons. P-value less than 0.05 considered statistically significant and results are expressed as the means ± SEM.

## Results

### Passive avoidance test

**Fig. 2:**
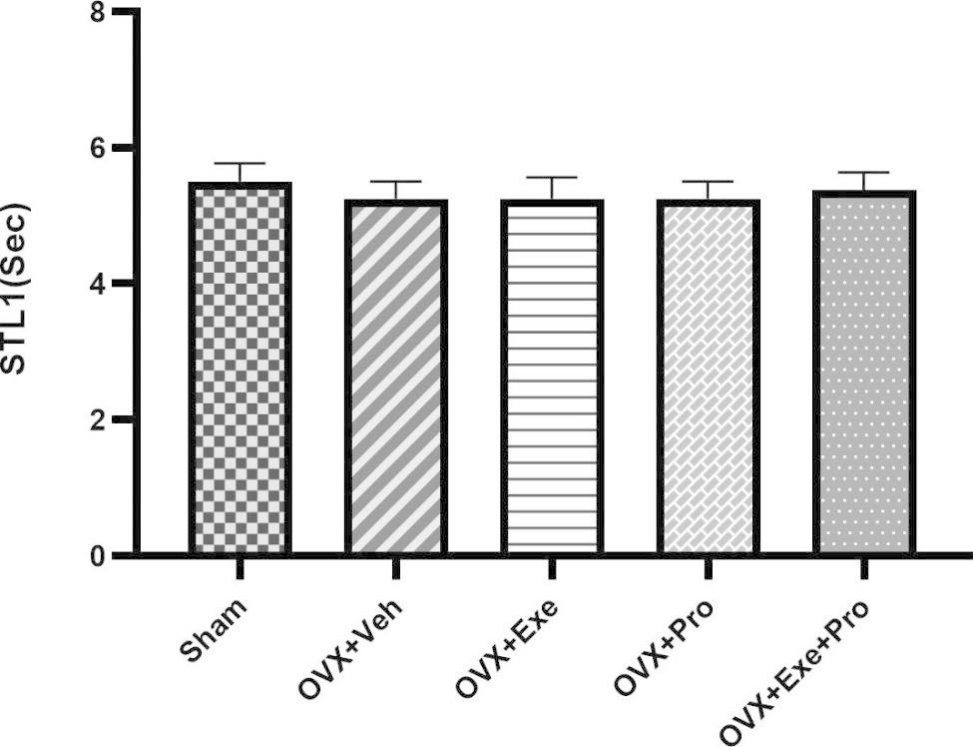
The effect of exercise, probiotic, and combination of both on step - through dark latency in day 1 (STL1). recorded for 20 s. OVX + Veh: Control group received of drinking water. OVX + Exe: high-intensity interval training group; OVX + Pro: consumption probiotic group; OVX + Exe + Pro: consumption probiotic with high-intensity interval training group. Kruskal-Wallis test followed by non-parametric Mann-Whitney test. Values are reported as the mean ± SEM, P - value less than 0.05, n = 8 rats per group

At the end of the training, the results of Kruskal-Wallis test indicated a significant difference between groups in STL2 (P = 0.02, df = 4, x2 = 10.793), TSD (P = 0.004, df = 4, x2 = 15.173) and frequency of crossing (P = 0.002, df = 4, x2 = 17.124), but not in STL1(P = 0.994, df = 4, x2 = 0.220, Fig. 2).

**Fig. 3:**
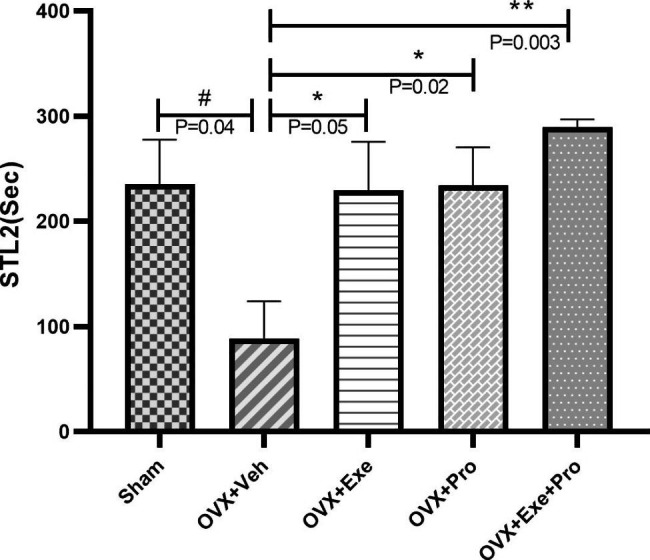
The effect of exercise, probiotic, and combination of both on step-through dark latency in day 2 (STL2). recorded for 300 s. OVX + Veh: Control group received of drinking water. OVX + Exe: high-intensity interval training group; OVX + Pro: consumption probiotic group; OVX + Exe + Pro: consumption probiotic with high-intensity interval training group. Kruskal-Wallis test followed by non-parametric Mann-Whitney test. Values are reported as the mean ± SEM, P - value less than 0.05, n = 8 rats per group. *P ≤ 0.05, **P ≤ 0.01, ***P ≤ 0.001 versus OVX + Veh group. #P ≤ 0.05, ##P ≤ 0.01, ###P ≤ 0.001 versus Sham group

In comparison with sham group, OVX + Veh showed significant difference in STL2 (P = 0.048, Z=-1.973, Wilcoxon = 50, Man-W = 14). Also it was a significant difference between the groups of OVX + Exe (P = 0.055,Z=-1.920, Wilcoxon = 50.5, Man-W = 14.5), OVX + Pro (P = 0.020,Z=-2.318, Wilcoxon = 46.5, Man-W = 10.5) and OVX + Exe + Pro (P = 0.003,Z=-2.960, Wilcoxon = 41, Man-W = 5) compared with OVX + Veh. There was no significant difference between OVX + Exe + Pro and monotherapy either by exercise or probiotic (p > 0.05, Fig. 3).

**Fig. 4:**
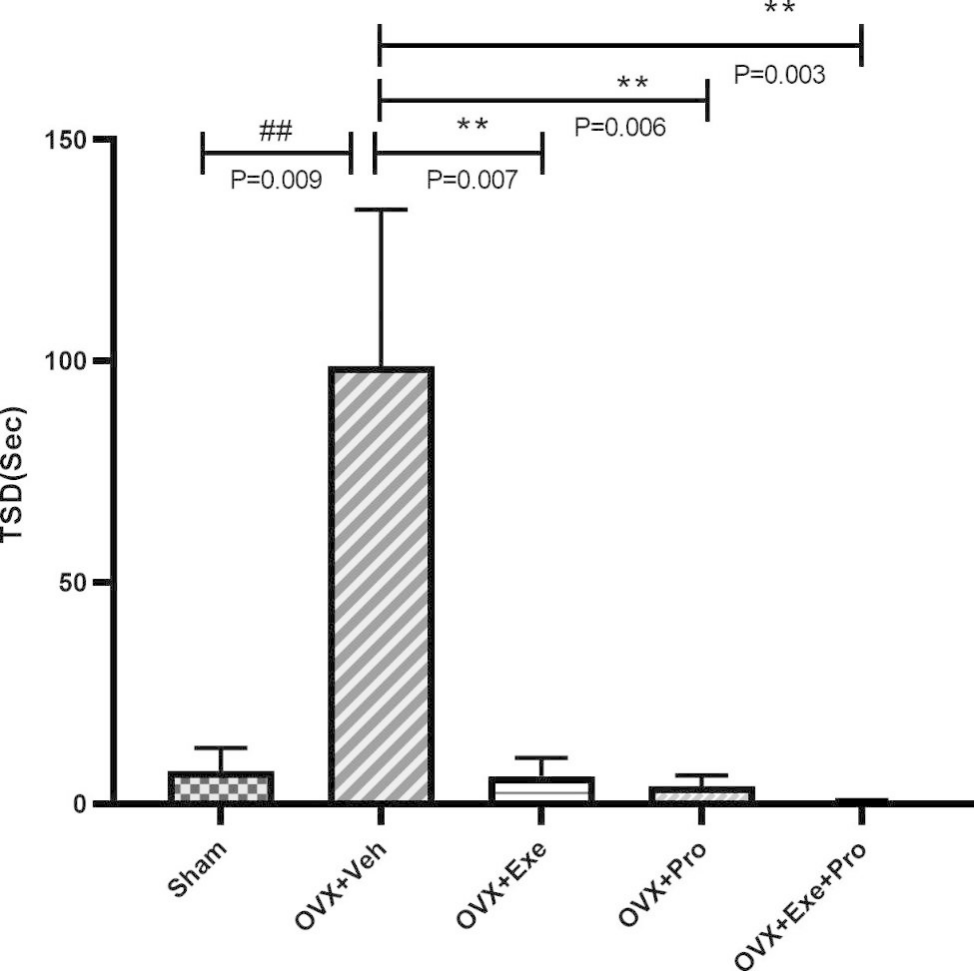
The effect of exercise, probiotic, and combination of both on total time spent in the dark compartment TSD. recorded for 300 s. OVX + Veh: Control group received of drinking water. OVX + Exe: high-intensity interval training group; OVX + Pro: consumption probiotic group; OVX + Exe + Pro: consumption probiotic with high-intensity interval training group. Kruskal-Wallis test followed by non-parametric Mann-Whitney test. Values are reported as the mean ± SEM, P - value less than 0.05, n = 8 rats per group. *P ≤ 0.05, **P ≤ 0.01, ***P ≤ 0.001 versus OVX + Veh group. #P ≤ 0.05, ##P ≤ 0.01, ###P ≤ 0.001 versus Sham group

Also significant difference was found in TSD between OVX + Veh group compared with Sham (P = 0.009,Z=-2.631, Wilcoxon = 44, Man-W = 8). The groups of OVX + Exe (P = 0.007,Z=-2.688, Wilcoxon = 43.5, Man-W = 7.5), OVX + Pro (P = 0.006,Z=-2.750, Wilcoxon = 42.5, Man-W = 6.5) and OVX + Exe + Pro (P = 0.003,Z=-2.962, Wilcoxon = 41, Man-W = 5) showed significant difference in in comparison with OVX + Veh. There was no significant difference between the latest three groups (p > 0.05, Fig. 4).

**Fig. 5:**
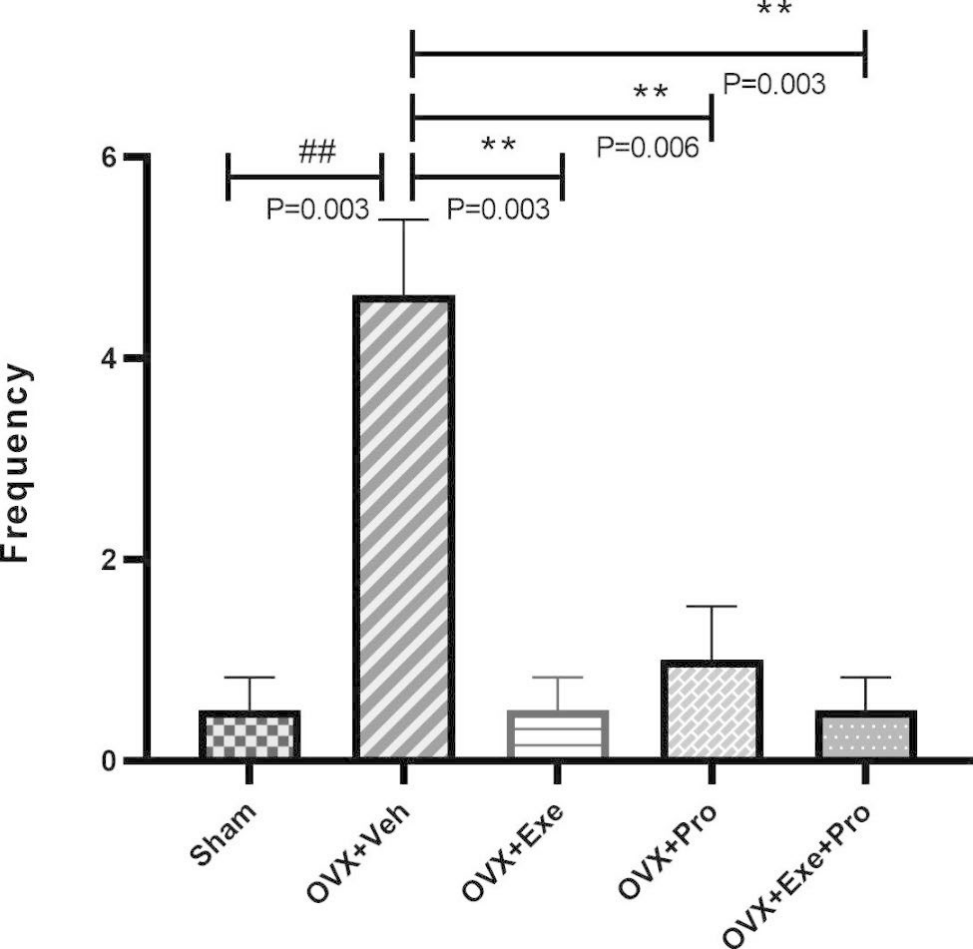
The effect of exercise, probiotic, and combination of both on frequency of entering into the dark compartments. recorded for 300 s. OVX + Veh: Control group received of drink; OVX + Exe: high-intensity interval training group; OVX + Pro: consumption probiotic group; OVX + Exe + Pro: consumption probiotic with high-intensity interval training group. Kruskal-wallis test followed by non-parametric mann-whitney test. Values are reported as the mean ± SEM, P - value less than 0.05, n = 8 rats per group. *P ≤ 0.05, **P ≤ 0.01, ***P ≤ 0.001 versus OVX + Veh group. #P ≤ 0.05, ##P ≤ 0.01, ###P ≤ 0.001 versus Sham group

Finally, Mann-Whitney test indicated a significant difference in frequency between OVX + Veh group compared with Sham. Significant difference was found between OVX + Exe group, OVX + Exe + Pro group (P = 0.003,Z=-2.970, Wilcoxon = 41, Man-W = 5) and OVX + Pro groups (P = 0.006,Z=-2.765, Wilcoxon = 42.5, Man-W = 6.5) compared with OVX + Veh. No significant difference was observed between the monotherapy and co-treatment of exercise and probiotics three groups (Fig. 5).

### Biochemical results

**Fig. 6:**
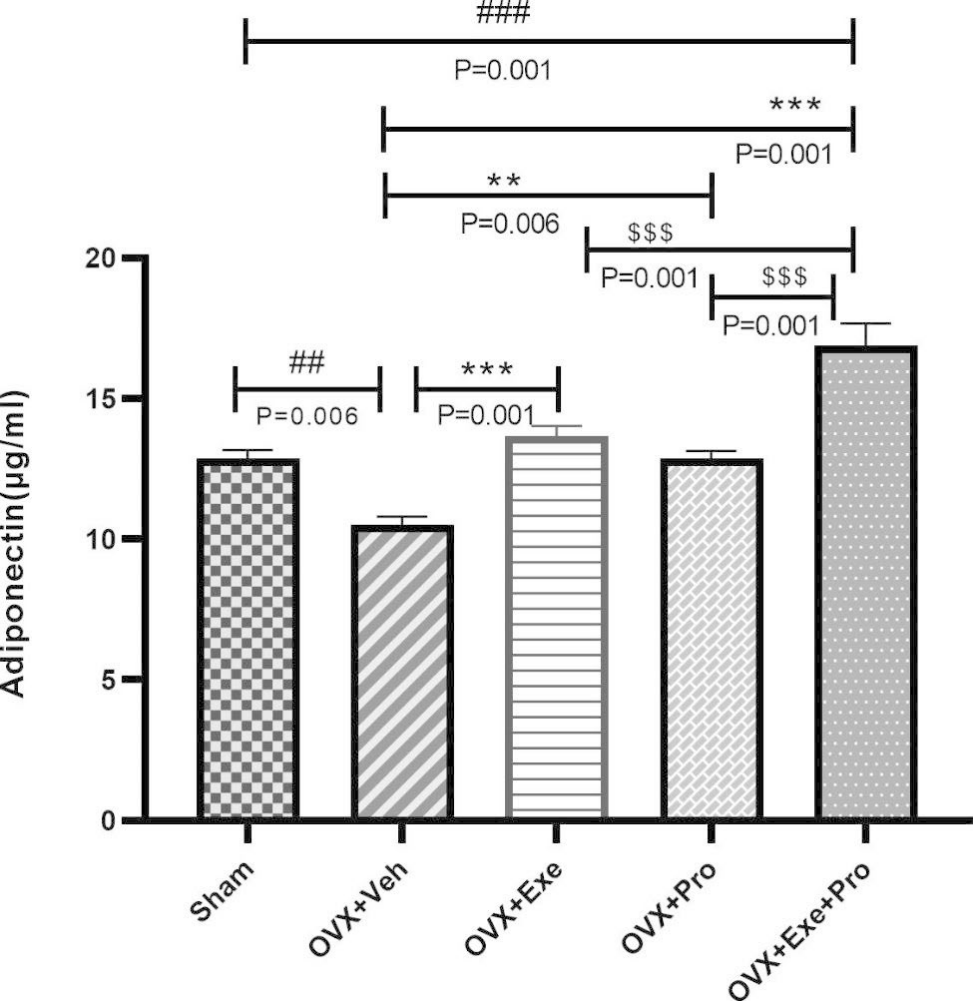
The effect of exercise, probiotic, and combination of both on adiponectin. OVX + Veh: Control group received of drinking water; OVX + Exe: high-intensity interval training group; OVX + Pro: consumption probiotic group; OVX + Exe + Pro: consumption probiotic with high-intensity interval training group. One-way analysis of variance test followed by Tukey’s post hoc test. Values are reported as the mean ± SEM, n = 8 rats per group.*P ≤ 0.05, **P ≤ 0.01, ***P ≤ 0.001 versus OVX + Veh group. #P ≤ 0.05, ##P ≤ 0.01, ###P ≤ 0.001 versus sham group. $P ≤ 0.05, $$P ≤ 0.01, $$$P ≤ 0.001 versus OVX + Exe + Pro group

One-way ANOVA test showed significant differences in serum adiponectin level F (4,35) = 26.061, p = 0.001), glucose (F (4,35) = 11.771, p = 0.001), insulin (F(4,35) = 7.750, p = 0.001), HOMA-IR (F(4,35) = 13.365, p = 0.001). Tukey test pairwise comparisons showed 18% decrease in serum adiponectin level for OVX + Veh compared with Sham group (p = 0.006). In contrary, it was elevated by 60% (OVX + Exe + Pro p = 0.001), 30% (OVX + Exe, p = 0.001), and 22% in (OVX + Pro, p = 0.006) compared with the OVX + Veh group. Also, significant difference was found in adiponectin level for OVX + Exe + Pro compared with OVX + Pro and OVX + Exe (p = 0.001, Fig. 6).

**Fig. 7:**
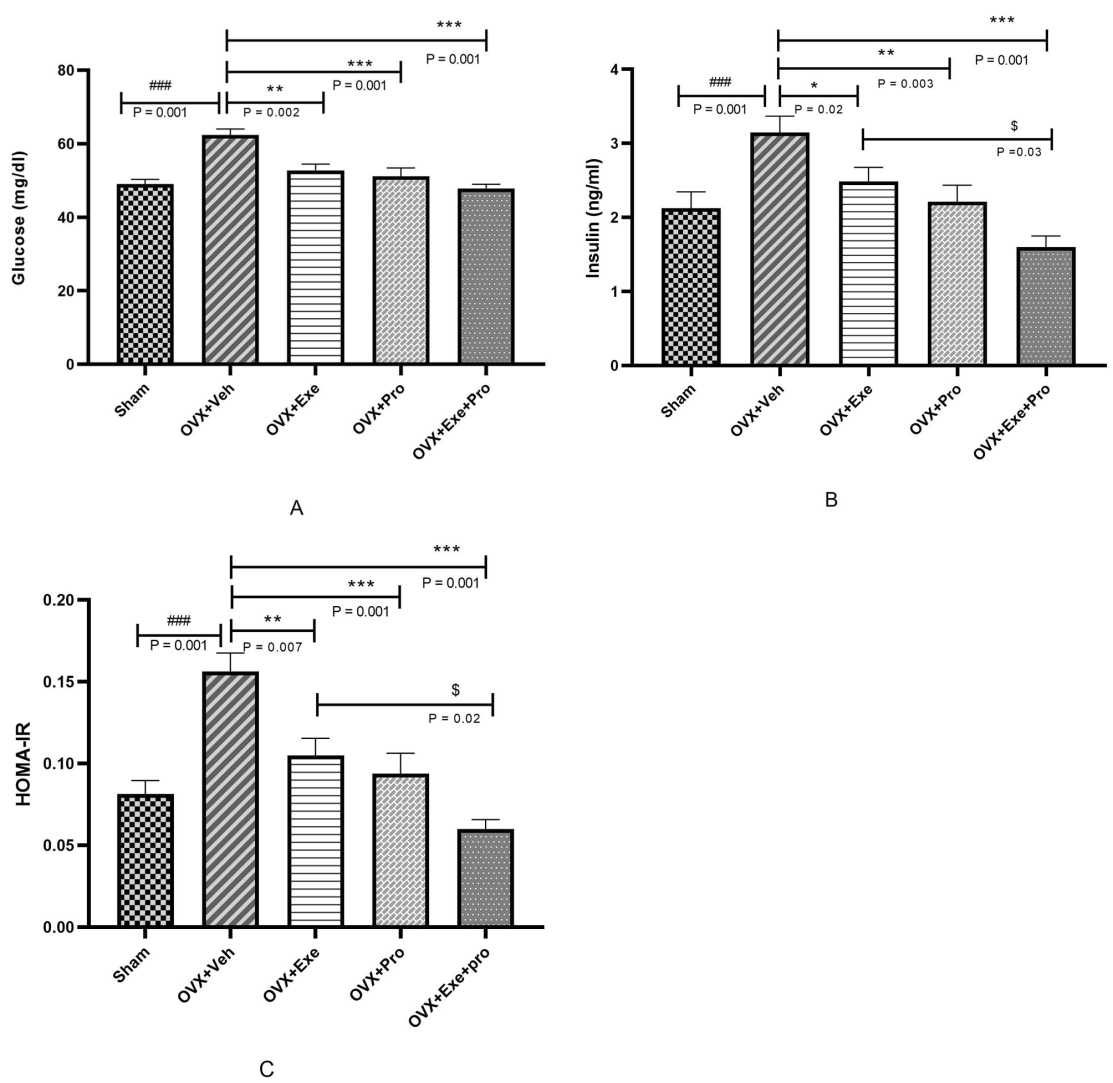
The effect of exercise, probiotic, and combination of both on (**A**) Glucose, (**B**) Insulin, (**C**) HOMA-IR. HOMA-IR: homeostasis model assessment of insulin resistance; OVX + Veh: Control group received of drink; OVX + Exe: high-intensity interval training group; OVX + Pro: consumption probiotic group; OVX + Exe + Pro: consumption probiotic with high-intensity interval training group. One-way analysis of variance test followed by Tukey’s post hoc test. Values are reported as the mean ± SEM, n = 8 rats per group.*P ≤ 0.05, **P ≤ 0.01, ***P ≤ 0.001 versus OVX + Veh group. #P ≤ 0.05, ##P ≤ 0.01, ###P ≤ 0.001 versus Sham group. $P ≤ 0.05, $$P ≤ 0.01, $$$P ≤ 0.001 versus OVX + Exe + Pro group

Glucose was increased by 27% in OVX + Veh compared with Sham (p = 0.001), and then it was decreased by 23% in (OVX + Exe + Pro, p = 0.001), 15% (OVX + Exe, p = 0.002) and 18% (OVX + Pro, p = 0.001, Fig. 7A).

In addition, insulin significantly increased by 48% in OVX + Veh compared with Sham (p = 0.001), and decreased by 49% in OVX + Exe + Pro (p = 0.001), 20% in OVX + Exe (p = 0.02) and 29% in OVX + Pro (p = 0.003) compared with the OVX + Veh group (Fig. 7B).

Finally 89% elevation in HOMA-IR was found in OVX + Veh compared with Sham (p = 0.001), while it was reduced in OVX + Exe + Pro (60%, p = 0.001), OVX + Exe (32%, p = 0.007), OVX + Pro (41%, p = 0.001) compared with the OVX + Veh group (Fig. 7C).

## Discussion

The findings of the present study showed memory impairment in MetS model of rats which was induced by ovariectomy surgery in line with the previous reports [11, 34]. Memory impairment in our study was evident by spending %33 of total time in the dark compartment compared with sham which spent only %2, and also shortening of latency to enter to the dark room. Also ovariectomy caused central adiposity, hyperglycemia, high serum insulin representing insulin resistance in agreement with our previous report [35].

It has previously reported that estrogen withdrawal leads to the metabolic syndrome [36], increase in systemic inflammatory cytokines [26],and memory impairment [11]. Estrogen impacts brain metabolic activity and redox status [37], and enhances hippocampal memory consolidation [38] via extracellular signal-regulated kinase/mitogen activated protein kinase (ERK/MAPK) and cyclic AMP response element binding protein (CREB) [39].

On the other hand, our menopause model of rats showed significant insulin resistance and adiposity after the end of three months. Insulin resistance, in turn promoted the development of cognitive dysfunction because of insulin signaling impairment [40]. Under normal conditions, the brain utilizes glucose in a 17β–estradiol-dependent way, however during menopause estrogen regulation of metabolic pathways are weakened, and cerebral glucose metabolism is declined, and this, shifts the metabolism toward mitochondrial utilization of ketone bodies, lipids and amino acids [41]. Brain glucose hypometabolism, mitochondrial dysfunction and reduced oxidative phosphorylation may cause formation of AD plaques [41]. Moreover, decrease in sex hormone levels is paralleled by an increase in oxidative stress in female brains which might be the beginning of inflammation and AD [37].

In addition, insulin resistance activates glycogen synthase3 (GSK3), which increases amyloid beta (Aβ) production and tau phosphorylation; two abnormal proteins responsible for cognition impairment and pathological features of AD. Hyperinsulinemia also causes cerebrovascular and blood brain barrier (BBB) dysfunction which also result in synaptic dysfunction and cognition impairment. On the other hand, pro-inflammatory cytokines and adipokines secreted from accumulated visceral fats cause malfunctions in nervous system [42].

Another part of our experiment showed that, chronic HIIT and simultaneous consumption of native potential probiotic bacteria improved retention and retrieval of associative memory, reduced blood glucose, insulin, HOMA IR and increased adiponectin levels in rats with menopause.

Regular physical activity has been known to improve learning and memory in MetS model of rats by reducing oxidative stress and inflammation, increasing BBB permability [43], and also increasing the levels of anti-inflammatory cytokines [44]. Some studies believe that, neurotrophic factors, especially brain derived neurotrophic factor (BDNF), are elevated after physical activities. BDNF is a protein expressed in the central nervous system and plays pivotal role in neural survival, synaptic plasticity, and neurogenesis [45]. In fact, skeletal muscles produce irisin, and this protein induces BDNF release from neurons [45]. Finally, sustained levels of BDNF during exercise, have important roles in cognition through stimulating long-term potentiation, protein phosphorylation, synaptic regeneration, and finally memory improvement in healthy and AD model of rats [46].

Also, irisin helps to brown the white adipose tissue and increasing thermogenesis, which consequently promotes insulin sensitivity. Furthermore, BDNF improves insulin sensitivity via increasing glucokinase activity and reducing hepatic gluconeogenesis [47].

In addition, our finding revealed a significant increase in serum adiponectin levels. This adipokine as an anti-inflammatory peptide treat metabolic syndromes via inducing insulin sensitivity, anti-inflammatory and anti-oxidative effects [43]. HIIT increases the transport of adiponectin across the BBB [43], then adiponectin binds with the receptor AdipoR1, and activates the AMP-activated protein kinase (AMPK) pathway, and increases neuronal insulin sensitivity, Besides, adiponectin binding with AdipoR2, stimulates the neural plasticity through the activation of the peroxisome proliferator-activated receptor alpha (PPARα) pathway and inhibits oxidative stress [23]. In contrary reduced adiponectin levels in the group of ovariectomized rats contribute to the deregulated glucose metabolism and mitochondrial dysfunction observed in the brain of AD [48]. Adiponectin can easily cross the BBB, and binds with adiponectin receptor1 and 2 (AdipoR1, AdipoR2), and T-cadherin in the hippocampus, cortex and hypothalamus regions of the brain and act as a neuroprotective peptide[24].

The group consumed probiotic for 8 weeks showed improvement in memory and HOMA-IR as well. Probiotic Lactobacillus reported to suppress hippocampal inflammatory genes expression and oxidative stress related genes [21, 49], whereas accumulation of intestinal bacteria secretes large amounts of amyloid β, lipopolysaccharide, and pre-inflammatory cytokines associated with AD pathogenesis [32].

Furthermore, co treatment of exercise training and probiotic for 8 weeks in the present study, synergistically facilitated memory storage and retrieval, and represents both neurotrophic an metabotrophic effects in menopause-induced metabolic syndrome model of rats.

## Conclusion

Our findings suggest that chronic high intensity interval training combined with native lactobacillus probiotic supplementation for 8 weeks alleviate associative memory deficit and insulin resistance partly via adiponectin.

## Data Availability

Data will be available on request from correspondent autor.
